# Child Psychomotricity: Development, Assessment, and Intervention

**DOI:** 10.3390/children10101605

**Published:** 2023-09-26

**Authors:** Ana Rita Matias, Gabriela Almeida, Guida Veiga, José Marmeleira

**Affiliations:** Comprehensive Health Research Centre (CHRC), Department of Sport and Health, School of Health and Human Development, University of Évora, 7004-516 Évora, Portugal; gsna@uevora.pt (G.A.); gveiga@uevora.pt (G.V.); jmarmel@uevora.pt (J.M.)

Psychomotricity addresses the interactions between psychic functions, motor (and biological) functions, and motor behavior (gestures, posture, attitude, physical activity, and motor skills). The theoretical foundations of psychomotricity are based on neuroscience, psychogenetics, cognitive psychology, and psychoanalysis [1], entailing a relational and mind–body perspective [2]. Psychomotricity helps individuals access their relational, symbolic, and emotional capacities through movement, action, and relation. Psychomotricity gives comprehensive care to individuals across their lifespan, either in typical or atypical development [2].

The body is at the center of Psychomotricity. Indeed, harmonious development requires identifying, differentiating, individualizing, and building a body envelope, as well as inhabiting one’s body. By receiving and processing the person’s tonic and emotional expression, the psychomotor therapist allows the individual to sense, reflect, and transform their body–mind experiences within a safe and transformative atmosphere provided by the therapeutic and bodily relationship [3]. In other words, the psychomotor therapist is specialized in creating synergies, promoting the expression and regulation of sensory–motor and mental functions within an emotional context [4], and contributing to improving well-being.

In recent years, the practice of psychomotricity has increased and widened its scope, addressing new human and social needs [5]. It is a profession with enormous recognition from the civil, professional, and scientific community. Such an increase requires more research to leverage scientific dissemination in psychomotricity [6] and help inform psychomotor therapists, other clinicians, researchers, and policymakers.

Driven by this purpose, this Special Issue intended to gather more scientific evidence on psychomotricity, focusing on psychomotor development, assessment instruments, and intervention practices. 

As can be noticed while reading the present Special Issue, “Child Psychomotricity: Development, Assessment, and Intervention”, a particular emphasis was given to the topic of assessment, both at the scales and skills level, with a specific focus on handwriting at different ages. Also, some authors presented their research on psychomotor intervention, covering aspects such as guidelines for autism spectrum disorder (ASD), perceptions of education teachers regarding psychomotor skills in the educational context, or observational studies of children’s behavior in the playground. Moreover, this Special Issue also addresses children with special needs, such as children who are deaf or hard of hearing.

Assessment is a critical stage of the psychomotor intervention, helping to know the person and supporting the definition of the diagnosis and the subsequent intervention planning and evaluation. Indeed, when it is impossible to establish a precise diagnosis, assessment supports the therapist in defining an individual’s clinical profile [1] and outlining a therapeutic action plan for intervention.

The interest in cross-cultural assessment instrument adaptation is a factor for cross-cultural research and educational and clinical practice, with increasing scientific attention [7]. Amorim et al. [8], Delgado et al. [9], and Samadi et al. [10] present us with three studies focused on the psychometric properties of three instruments of assessment, the first two for Portuguese children and the third one for Kurdish. Amorim et al. [8] contributed to a deeper understanding of typical development using a psychomotor assessment instrument: Battery for Neuropsychomotor functions evaluation (NP-MOT), which was also applied to children with developmental disorders. Similarly, Samadi et al. [10] highlighted the importance of the Gilliam autism rating scale—third edition (GARS-3)—widely used to diagnose children with ASD, developing their research in typically developing children and children with intellectual disabilities with communication disorders. Delgado et al. [9] take us back to the educational context by presenting the Writing Readiness Inventory Tool in Context (WRITIC), an instrument to assess children’s level of handwriting readiness between 4 and 6 years. Although the presented instruments showed a valid and reliable profile, the authors conclude that more studies are needed to consolidate the results obtained. 

Coradinho et al. [11] examined the relation between process and product measures during handwriting in second graders. Their results are the first step towards creating normative values in the process characteristics of handwriting, contributing to dysgraphia early identification. Also, Hen-Herbst and Rosenblum [12] investigated the relations between handwriting measures, motor-related daily performance, and health-related quality of life in adolescents with and without dysgraphia. The authors showed the negative effects that a dysgraphia diagnosis might have, reducing adolescents’ perception of quality of life, therefore impacting their social–emotional well-being [13].

The psychomotor therapist plays a vital role at school when framed within a systemic intervention to support the school-aged individual [1]. In this Special Issue, the school context has particularly addressed both the level of teacher’s perceptions [14,15] and the psychomotor practices in children with ASD [15]. Rojo-Ramos et al. [14] analyzed teachers’ perceptions about the needs and current state of psychomotor competencies in the educational context of Extremadura schools, comparing the information in both rural and urban areas. The authors concluded that teachers lack training, materials, and facilities for effective psychomotor intervention, with discrepancies between urban and rural areas. Similarly, the article by Alkahtani [16] studied teachers’ perceptions of their students diagnosed with emotional and behavioral disorders and reported insufficient teacher training regarding this matter, which could lead to teachers’ negative attitudes towards these children. Finally, Frazão et al. [15] aimed to generate expert consensus regarding psychomotor intervention guidelines for 3- to 6-year-old children diagnosed with ASD. Their consensus-based approach resulted in 88 guidelines, where approximately half reached more than 95% agreement. This was an essential contribution to supporting psychomotor therapist intervention with autistic children.

Research on play and the outdoors became even more relevant in the last few years. Researchers and educators became aware that the tendency to reduce the involvement of children in free play and outdoor time compromises children’s psychosocial and motor development [17]. In this Special Issue, the space outside the classroom was also addressed, either by focusing on play-based intervention in the outdoors [18] by examining play behaviors of children deaf and hard of hearing (DHH) in the outdoor school playground [19] or by developing a sensor-based instrument to analyze children’s behaviors and its relationship with the playground affordances. Specifically, Pereira et al. [18] analyses of children’s play patterns during ten play sessions in a primary school showed that all four types of materials (tarpaulin/fabrics, cardboard boxes, plastic crates, and plastic tubes) have the same importance for these children. Moreover, Silva et al. [19] showed that DHHs do not play as much as their hearing peers and that interventions are needed to transform the physical environment into a more friendly and socially facilitating context. Finally, the innovative data-driven approach presented by Nasri et al. [20] has great potential to understand children’s interactions with schoolyard environments and inform psychomotor therapists about their children and the effects of their interventions.

Closing this Special Issue, it is clear that the articles included contributed to consolidating the research in child psychomotricity, reinforcing the importance of psychomotor practice. 
